# LPS-Induced Low-Grade Inflammation Increases Hypothalamic JNK Expression and Causes Central Insulin Resistance Irrespective of Body Weight Changes

**DOI:** 10.3390/ijms18071431

**Published:** 2017-07-04

**Authors:** Rodrigo Rorato, Beatriz de Carvalho Borges, Ernane Torres Uchoa, José Antunes-Rodrigues, Carol Fuzeti Elias, Lucila Leico Kagohara Elias

**Affiliations:** 1 Department of Physiology, Ribeirao Preto Medical School, University of Sao Paulo, Sao Paulo 14049-900, Brazil; borgesbc@gmail.com (B.d.C.B.); ernane_uchoa@yahoo.com.br (E.T.U.); jantunesr@gmail.com (J.A.-R.); 2 Department of Physiological Sciences, State University of Londrina, Londrina 86057-970, Brazil; 3 Department of Molecular and Integrative Physiology, University of Michigan, Ann Arbor, MI 48109-5622, USA; cfelias@med.umich.edu

**Keywords:** LPS tolerance, hypothalamic inflammation, insulin resistance, pJNK

## Abstract

Metabolic endotoxemia contributes to low-grade inflammation in obesity, which causes insulin resistance due to the activation of intracellular proinflammatory pathways, such as the c-Jun N-terminal Kinase (JNK) cascade in the hypothalamus and other tissues. However, it remains unclear whether the proinflammatory process precedes insulin resistance or it appears because of the development of obesity. Hypothalamic low-grade inflammation was induced by prolonged lipopolysaccharide (LPS) exposure to investigate if central insulin resistance is induced by an inflammatory stimulus regardless of obesity. Male Wistar rats were treated with single (1 LPS) or repeated injections (6 LPS) of LPS (100 μg/kg, IP) to evaluate the phosphorylation of the insulin receptor substrate-1 (IRS1), Protein kinase B (AKT), and JNK in the hypothalamus. Single LPS increased the expression of pIRS1, pAKT, and pJNK, whereas the repeated LPS treatment failed to recruit pIRS1 and pAKT. The 6 LPS treated rats showed increased total JNK and pJNK. The 6 LPS rats became unresponsive to the hypophagic effect induced by central insulin administration (12 μM/5 μL, ICV). Prolonged exposure to LPS (24 h) impaired the insulin-induced AKT phosphorylation and the translocation of the transcription factor forkhead box protein O1 (FoxO1) from the nucleus to the cytoplasm of the cultured hypothalamic GT1-7 cells. Central administration of the JNK inhibitor (20 μM/5 μL, ICV) restored the ability of insulin to phosphorylate IRS1 and AKT in 6 LPS rats. The present data suggest that an increased JNK activity in the hypothalamus underlies the development of insulin resistance during prolonged exposure to endotoxins. Our study reveals that weight gain is not mandatory for the development of hypothalamic insulin resistance and the blockade of proinflammatory pathways could be useful for restoring the insulin signaling during prolonged low-grade inflammation as seen in obesity.

## 1. Introduction

Chronic low-grade inflammation is associated with leptin and insulin resistance, which contributes to the establishment of obesity and its comorbidities, such as type 2 diabetes, cancer, and cardiovascular diseases [1]. We have previously demonstrated that low-grade inflammation induced by repeated injections of the Gram-negative bacterial lipopolysaccharide (6 daily doses of LPS) induces tolerance to the hypophagic effect of the endotoxin [2,3]. The endotoxin failure to reduce food consumption and body weight in LPS-tolerant rats is associated with unresponsiveness to leptin to phosphorylate the signal transduction and activator of transcription 3 (pSTAT3) protein in the hypothalamic nuclei crucial for the control of the energy homeostasis, such as the paraventricular (PVN) and arcuate (ARC) nuclei [2]. Despite the unresponsiveness to leptin in rats treated with six daily doses of LPS, these animals do not show body weight change as opposed to an increase of body weight and fat mass seen in rats after seven days of a high fat diet regimen [4]. Hence, repeated exposure to LPS might be a useful approach to dissociate the impact of the low-grade inflammation that precedes the increased adiposity signals on the development of peripheral and hypothalamic resistance seen in obesity. 

The central infusion of insulin induces a negative energy balance comparable to the central administration of leptin [5]. The appetite-suppressive and weight-reducing effects of insulin have been shown in both rodents and primates [6,7]. These effects are supported by the presence of the insulin receptor (IR) and the expression of intracellular components of the insulin signaling, the insulin receptor substrate 1 (IRS-1), and the phosphoinositide 3-kinase (PI3K) pathway-induced proteins in the hypothalamus [8,9,10,11]. The PI3K pathway is recruited by both insulin and leptin in the control of energy homeostasis. Pharmacological blockade of PI3K prevents the suppression of food consumption induced by intracerebroventricular (ICV) administration of both leptin and insulin [10,12]. The PI3K pathway also plays a role in the hypophagia acutely induced by LPS [13]. However, the impact of low-grade inflammation induced by prolonged LPS exposure on central insulin signaling has not been addressed.

Hypothalamic insulin resistance may be evoked by inflammatory stimuli [14,15] via the activation of intracellular proinflammatory pathways that increase the expression of intermediate proteins like Jun NH_2_-terminal kinase (JNK), protein tyrosine phosphatase 1B (PTP1B), inhibitor of nuclear factor kappa kinase (IKK), and endoplasmic reticulum stress [16,17,18,19]. Increased expression of these intermediates of cytokine signaling induces insulin resistance via the alteration of IR activation and expression, as well as by increasing the phosphorylation of the IRS-1 in serine residues, which impairs insulin signal transduction [14,15]. The JNK is a serine kinase activated by cytokines and free fatty acids [15,19,20] and increased JNK activity in the hypothalamus was observed in High fat diet (HFD) fed animals [16,21]. Reinforcing the role of JNK in the development of hypothalamic insulin resistance in HFD fed rodents, mice with JNK1 deficiency in the brain exhibit improved insulin sensitivity in both central and peripheral tissues, preventing adipose tissue dysfunction and hepatic steatosis under HFD feeding [21].

Despite the evident association between obesity and insulin resistance and the activation of the JNK-mediated inflammatory pathway, it remains unclear whether the activation of this pathway precedes the development of obesity in HFD fed animals. To circumvent this challenge, we used prolonged LPS treatment to induce low-grade inflammation and to investigate whether the repeated exposure to endotoxins might increase JNK phosphorylation and cause hypothalamic insulin resistance, independently on the development of obesity. To further address the effect of endotoxins on insulin signaling in neurons, we used cultured GT1-7 cells treated with LPS.

## 2. Results and Discussion

### 2.1. Acute, but Not Prolonged Endotoxemia, Promotes Insulin Secretion and Activates Insulin Signaling Cascades. Short and Long-Term LPS Exposure Increases N-Terminal Kinase (JNK) Phosphorylation in the Hypothalamus

**Figure 1:**
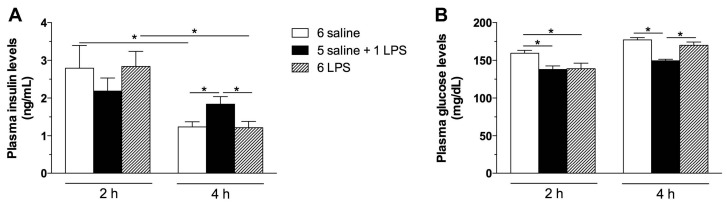
Acute, but not prolonged endotoxemia, increases plasma insulin and reduces glucose levels. Plasma insulin (**A**) and glucose (**B**) levels in saline (6 saline), single (5 saline + 1 LPS, 100 μg/kg ip), or repeated lipopolysaccharide (6 LPS) treated-animals. The representative results of two independent experiments (*n* = 6–8/group) are shown with the measurements performed with samples from the same animal. One-way ANOVA, followed by the Fisher post hoc test were performed. Data are expressed as means ± SE. Differences were accepted as significant at * *p* < 0.05.

To evaluate whether long-term endotoxemia stimulates insulin secretion and insulin signaling in the hypothalamus, rats were treated with repeated LPS injections in comparison with single LPS treatment. Plasma insulin levels were increased 4 h after single, but not repeated LPS treatment (Figure 1A). Coupled to this response, glucose levels were reduced in single LPS treated rats (Figure 1B) at that time. Insulin secretion following acute LPS administration has been previously described [22]. The release of insulin during the infection process is an adaptive response to the increased production of cytokines [22,23,24]. Because repeated exposure to endotoxins leads to a desensitization of the neuroendocrine and immunological systems [25], the unaltered insulin levels in the 6 LPS group, compared with the controls, indicates a tolerance to the LPS effects.

**Figure 2:**
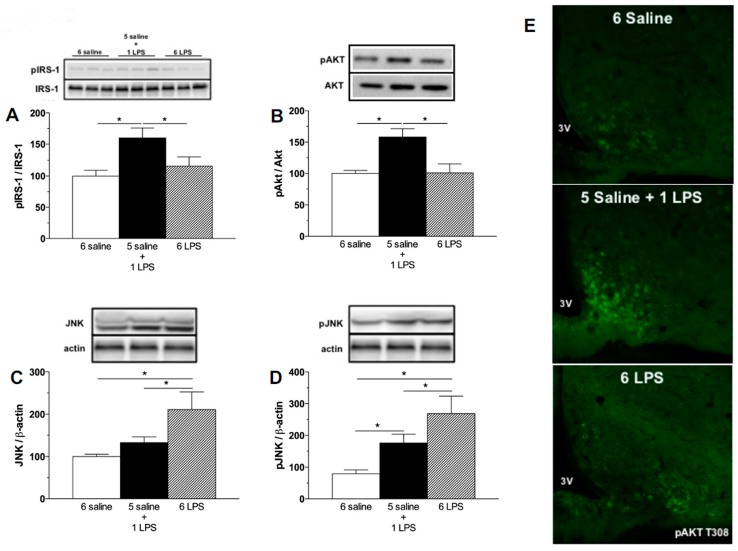
Acute endotoxemia increases phosphorylation of insulin signaling proteins whereas prolonged endotoxemia induces higher expression of the inhibitor of insulin cascade signaling, c-Jun N-terminal Kinase (JNK). Percentage of Phospho-Insulin receptor substrate-1 (pIRS1) (**A**), Phospho-Protein kinase B (pAKT) (**B**), JNK (**C**), and Phospho-c-Jun N-terminal Kinase (pJNK) (**D**) expression in the mediobasal hypothalamus of saline (6 saline), single (5 saline + 1 LPS, 100 ug/kg ip), or repeated LPS (6 LPS) treated-animals. The representative results of two independent experiments (*n* = 6–8/group) are shown with the measurements performed with samples from the same animal. One-way ANOVA, followed by Fisher post hoc test was performed. Data are expressed as means ± SE. Differences were accepted as significant at * *p* < 0.05; (**E**) representative photomicrographs showing the pAKT T308 expression (green) in neurons from the arcuate (ARC) nucleus of the hypothalamus of animals treated with 6 saline, 5 Saline + 1 LPS, and 6 LPS injections. 3V, third ventricle. Objective: 40×.

Evaluating the activation of insulin signaling, increased phosphorylation of the IRS-1 at the tyrosine 1222 residue and Protein kinase B (AKT) at the serine 473 and T308 residues in the hypothalamus of the single LPS group were observed, but not in repeated LPS treated rats (Figure 2A,B,E), confirming that acute LPS activates the insulin signaling pathways in the hypothalamus. A previous study from our group [13] reported the phosphorylation of AKT in leptin receptor-expressing neurons in the ARC of mice after acute LPS treatment. As leptin and insulin receptors partly colocalize in the ARC neurons [26], it is reasonable to postulate that an inflammatory stimulus is likely to increase phosphorylation of IRS-1/AKT proteins both in response to leptin and insulin. At present, it was not possible to determine if the single LPS treatment increases insulin signaling per se or if this is an indirect effect mediated by the LPS-induced cytokines, as the expression of Toll-like receptor 4 in neurons is not established [27]. The direct role of endotoxins in the activation of insulin signaling cascades deserves further investigation. Interestingly, as opposed to the 1 LPS group, 6 LPS treated rats did not show increased pIRS-1 and pAKT, supporting our hypothesis that prolonged endotoxemia induces hypothalamic failure to activate insulin signaling proteins.

Insulin resistance can be induced by the increased expression of several kinases that inhibit the eIRS-PI3K-AKT pathways, including JNK. JNK proteins are known to be recruited by LPS as well as during HFD feeding [16,28]. JNK activation increases spontaneous action potential firing of hypothalamic Agouti related peptide (AgRP) expressing neurons and induces both central and peripheral leptin resistance [29]. The phosphorylation of JNK impairs the phosphorylation of tyrosine residues of the IRS-1 and subsequently inhibits AKT activation by insulin in HFD fed rodents [16]. In the present study, prolonged LPS exposure increased both total JNK expression and JNK phosphorylation in the hypothalamus, whereas acute LPS treatment increased only Phospho-c-Jun N-terminal Kinase (pJNK) expression (Figure 2C,D). It is important to highlight that the increased pJNK expression induced by 6 LPS injection was significantly higher than that induced by the single LPS treatment (Figure 2D). Taken together, these findings suggest that hypothalamic unresponsiveness to insulin during a low-grade inflammation challenge could be due to a higher JNK activation. 

### 2.2. Hypophagic Effect of Central Insulin Injection Is Blunted in Acute and Prolonged Lipopolysaccharide—Treated Rats 

Hypothalamic leptin and insulin resistance are important in the pathophysiology of obesity after high fat feeding and they have been associated with hypothalamic inflammation. In HFD fed animals, the hypothalamic inflammation characterized by the production of cytokines and activation of the microglia precedes the weight gain, the peripheral fat accumulation, and the development of insulin resistance [4]. Insulin receptors are expressed in hypothalamic neurons of the melanocortin system [30] and insulin treatment increases proopiomelanocortin (POMC) and decreases Agouti-related peptide (AgRP)/neuropeptide Y (NPY) expression in the hypothalamus through the activation of the IRS-PI3K-AKT pathway [30,31,32], inhibiting feeding responses. Neuronal IR knockout mice are obese and sensitive to HFD [33], reinforcing the important role of central insulin action in the regulation of energy homeostasis. 

**Figure 3:**
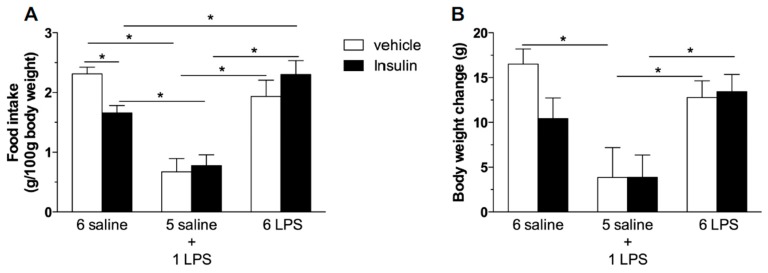
Prolonged endotoxemia induces resistance to the hypophagic effect of icv insulin treatment. Effect of icv injection of vehicle (saline, 5 μL) or insulin (12 μM in 5 μL) on food intake (**A**) and body weight gain (**B**) in saline (6 saline), single (5 saline + 1 LPS, 100 ug/kg ip), or repeated LPS (6 LPS) treated-animals (6–8 animals per group). The representative results of two independent experiments (*n* = 6–8/group) are shown. Two-way ANOVA, followed by the Fisher post hoc test were performed. Data are expressed as means ± SE. Differences were accepted as significant at * *p* < 0.05.

We then tested if prolonged endotoxemia could cause central insulin resistance independent of changes in the body weight and in the peripheral sensitivity to insulin. According to previous reports, ICV insulin administration causes hypophagia [6,7]. As expected, we reproduced these findings given that central insulin administration inhibited food intake in saline treated rats (Figure 3A). The single LPS group displayed a reduced food intake and weight gain (Figure 3B), which were not further reduced by central insulin treatment. This result indicates that acute LPS treatment recruits the signaling cascade common to insulin signaling, as seen by the increased IRS-1 and AKT phosphorylation after the single endotoxin injection. On the other hand, 6 LPS rats, which present no altered food intake and weight gain, were not sensitive to the hypophagic effect of central insulin, suggesting that LPS-tolerant rats are centrally resistant to insulin (Figure 3).

### 2.3. Prolonged LPS Treatment Blocks Insulin-Induced Protein Kinase B (AKT) Phosphorylation in the Culture of Neuronal Cells

**Figure 4:**
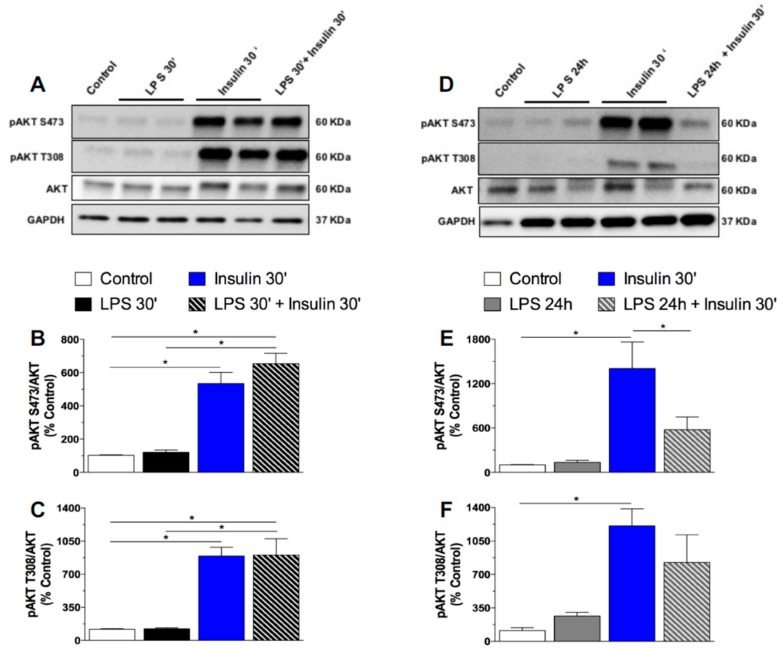
Prolonged exposure to LPS impairs insulin signaling in hypothalamic neurons in vitro. Percentage of pAKT S473 (**A**) and pAKT T308 (**B**) in the mouse GT1–7 cell line after saline (control), short (30′) LPS (1 μg/mL), insulin (100 mM/mL), or LPS (30′) + insulin treatment. Percentage of pAKT S473 (**E**) and pAKT T308 (**F**) in the mouse GT1–7 cell line after saline (control), prolonged (24 h) LPS (1 μg/mL), insulin (100 mM/mL), or LPS (24 h) + insulin treatment. In Vitro experiments were performed in 3–4 different assays. One-way ANOVA, followed by the Fisher post hoc test were performed. Data are expressed as means ± SE. Differences were accepted as significant at * *p* < 0.05.

**Figure 5:**
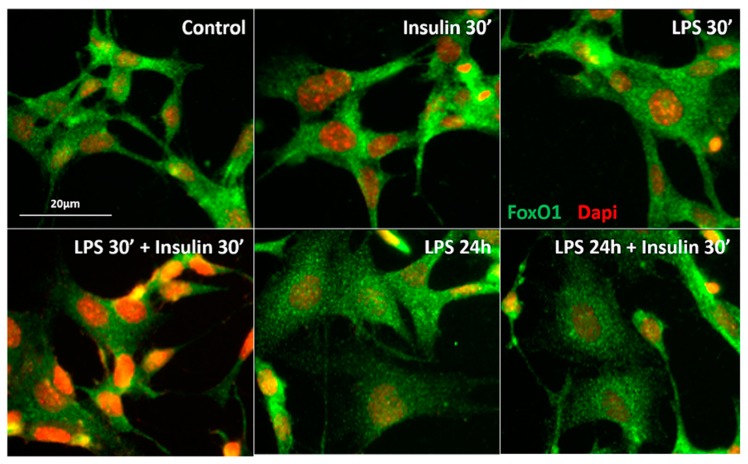
Prolonged LPS treatment impairs the ability of insulin to translocate Forkhead box protein O1 (FoxO1) from the nucleus to the cytoplasm. Representative photomicrograph of the qualitative FoxO1 immunostaning in the mouse GT1-7 cell line after short (30′) LPS (1 μg/mL), insulin (100 mM/mL), LPS (30′) + insulin, prolonged (24 h) LPS, or LPS (24 h) + insulin treatment. FoxO1 expression is in green and DAPI nuclear expression is in red. Scale bar: 20 μm.

As previously mentioned, the expression of toll-like receptors in neurons is undetermined [27]. However, it was reported that the GT1-7 cell line, derived originally from immortalized hypothalamic neurons, expresses toll like receptors and presents increased interleukins and tumor necrosis factor (TNF)-α mRNA expression after inflammatory stimulus with LPS [34]. To confirm whether long-term exposure to LPS impairs insulin signaling in neurons, we performed in vitro experiments in which we treated GT1-7 cells with LPS for short (30′) or long (24 h) periods, followed by treatment with insulin. LPS at both 30′ and 24 h did not induce pAKT expression in GT1-7 cells. As expected, cultured cells exhibited an increased AKT phosphorylation at both the S473 and T308 residues after insulin treatment. Short-term exposure to LPS (30 min) did not affect insulin-induced pAKT in these cells. Remarkably, prolonged LPS exposure (24 h) impaired the phosphorylation of AKT in both residues after insulin treatment (Figure 4). Activation of the IRS-PI3K-AKT pathway by insulin culminates with the sequestration of the Forkhead box protein O1 (FoxO1) into the cytoplasm, due to FoxO1 phosphorylation. FoxO1 mediates the anorectic effects of leptin and insulin in the ARC by regulating the transcription of POMC and AgRP [35]. We performed an in vitro qualitative study to evaluate the FoxO1 subcellular localization in GT1-7 cells under short- or long-term LPS exposure. In control cells, FoxO1 (green) is widely expressed in the whole cell. After insulin treatment, both control and LPS treated cells (30′) present a qualitatively higher FoxO1 expression predominantly located in the cytoplasm, as evidenced by a clear red DAPI nuclear staining. Short-term LPS treatment did not change FoxO1 expression/distribution in the GT1-7 cells, nor insulin-induced FoxO1 translocation from the nucleus to the cytoplasm. Interestingly, prolonged LPS treatment impaired the ability of insulin to induce the translocation of FoxO1 from the nucleus to the cytoplasm (Figure 5), evidencing the absence of insulin-induced phosphorylation and translocation of this protein to the cytoplasm. These data strongly suggest that low-grade inflammation induced by prolonged exposure to endotoxins impairs insulin signaling and leads to insulin resistant-like phenomena in the neurons.

### 2.4. Central JNK Inhibition Restores the Hypothalamic Insulin Responsiveness in Rats Exposed to Repeated LPS Injections

**Figure 6:**
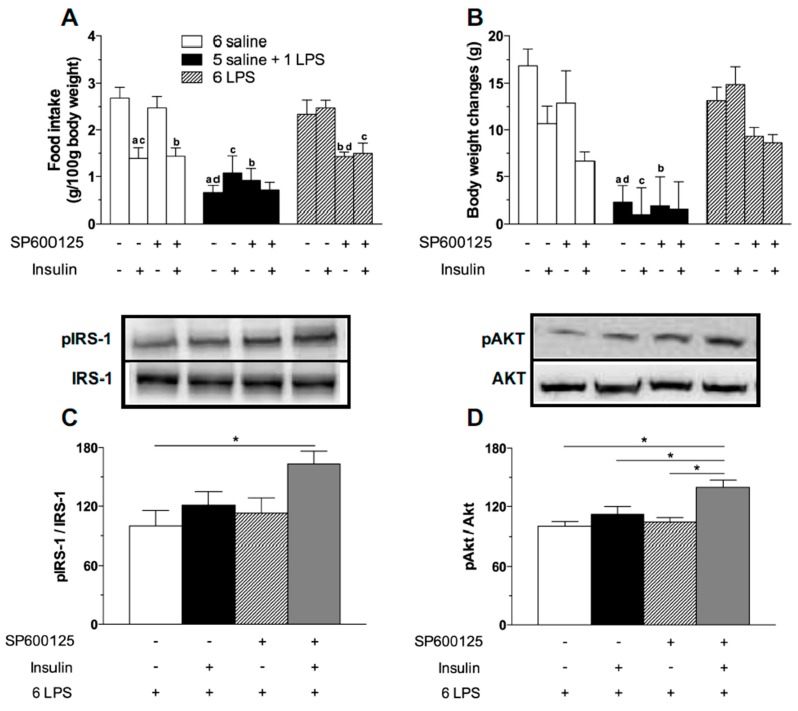
JNK inhibitor treatment restores the hypophagic effect of icv insulin treatment in 6 LPS-treated animals as well as restores the ability of icv insulin to phosphorylate IRS1 and AKT in 6-LPS treated rats. Effect of icv injection of vehicle (saline, 5 μL) or insulin (12 μM in 5 μL) on food intake (**A**) and body weight gain (**B**) in saline (6 saline), single (5 saline + 1 LPS, 100 ug/kg ip), or repeated LPS (6 LPS) treated-animals (6–8 animals per group) previously treated with icv injection of vehicle (saline, 5 μL) or SP600125 (20 μM in 5 μL). The representative results of three independent experiments (*n* = 6–8/group) are shown. Three-way ANOVA, followed by the Fisher post hoc test were performed. Data are expressed as means ± SE. Differences were accepted as significant at * *p* < 0.05. a vs. 6 saline + vehicle + saline, b vs. 6 saline + SP600125 + saline, c vs. 6 LPS + vehicle + Insulin, d vs. 6 LPS + vehicle + saline and e vs. 1 LPS + vehicle + Insulin. Graphs (**C**,**D**) show the percentage of pIRS-1 and pAKT expression, respectively, in the mediobasal hypothalamus of repeated LPS (6 LPS) treated-animals that received an icv injection of vehicle (saline, 5 μL) or insulin (12 μM in 5 μL) and were previously treated with an icv injection of vehicle (saline, 5 μL) or SP600125 (20 μM in 5 μL). The representative results of two independent experiments (*n* = 6–8/group) are shown with the measurements performed with samples from the same animal. One-way ANOVA, followed by the Fisher post hoc test were performed. Data are expressed as means ± SE. Differences were accepted as significant at * *p* < 0.05.

Since JNK causes insulin resistance and we have observed a higher expression of this kinase in the hypothalamus of 6 LPS treated rats, we investigated if we could alleviate the hypothalamic insulin resistance inhibiting the JNK activity. No effect of the selective JNK inhibitor SP600125 was observed in saline or single LPS treated animals (Figure 6A). Remarkably, JNK inhibition restored the hypophagic effect of LPS in 6 LPS tolerant rats. Additionally, there was no further hypophagia in 6 LPS animals treated with SP600125 after stimulation with central insulin (Figure 6A). Body weight was not affected by JNK inhibition (Figure 6B). The data support the hypothesis that JNK plays a role in the tolerance to the hypophagic effect of LPS during prolonged endotoxemia. 

To verify if the reversion of hypophagia in LPS tolerant rats after JNK inhibition was associated with a recovery of the hypothalamic ability to promote insulin signaling, we assessed the expression of pIRS1 and pAKT in the 6 LPS group. Insulin-induced phosphorylation of both IRS1 and AKT was higher in the 6 LPS rats treated with SP600125, compared with the other groups. Interestingly, in the 6 LPS group the JNK inhibitor restored the hypophagic effect of the endotoxin, despite no changes in the activation of insulin signaling proteins. Since it was recently demonstrated that JNK inhibition was able to sensitize leptin’s anorectic effect by increasing leptin-induced STAT3 activation and SOCS3 downregulation in the hypothalamus of DIO animals [36], we propose that the activation of alternative pathways not investigated in our study might account for the restoration of the hypophagia. Interestingly, the JNK inhibitor restored the insulin signaling in 6 LPS tolerant rats (Figure 6C,D), reinforcing the role of JNK in the development of insulin resistance during low-grade inflammation. Supporting our results, JNK1 ablation in the CNS improves hypothalamic and systemic insulin sensitivity in mice fed with HFD [21,37]. Administration of the JNK selective inhibitor SP600125 restored the insulin signaling in HFD obese rats [16]. Therefore, our study demonstrates the involvement of the intracellular pro-inflammatory JNK pathway in the development of hypothalamic insulin resistance during prolonged endotoxemia. Our data also suggest that insulin sensitivity can be restored through treatment with drugs that selectively inhibit this intracellular pro-inflammatory pathway.

## 3. Material and Methods 

### 3.1. Animals

Adult male Wistar rats weighing 220–250 g (Central Animal Facility of the University of Sao Paulo-Campus Ribeirao Preto) were individually housed under controlled light (12:12 h light-dark cycle; lights off at 06:00 p.m.) and temperature conditions (23 ± 1 °C), with free access to water and food, unless otherwise stated. Food consumption and body weight were recorded daily between 03:30–04:00 p.m. Rats were acclimated to the procedures of drug administration during the experiments by daily handling. 

All procedures for the care and use of animals were approved by the Ethical Committee for Animal Use of the Ribeirao Preto Medical School (027/2011, 28 March 2011).

### 3.2. Experimental Procedures

#### 3.2.1. Effect of Single or Repeated LPS Injections on Plasma Insulin and Glucose Levels, as Well as in the Hypothalamic Content of Insulin Signaling Proteins

Rats were assigned into three groups (*n* = 6–8/group): (1) saline once daily for 6 days (6 Saline), (2) saline once daily for 5 days and an injection of LPS on the 6th day (5 Saline + 1 LPS), and (3) LPS once daily for 6 days (6 LPS). Rats received saline (0.15 M NaCl, 1 mL/kg) or LPS (100 μg/kg, 1 mL/kg; Serotype 026:B6; Sigma, St. Louis, MO, USA) by an intraperitoneal (IP) injection at 04:00 p.m. On the 6th day of injection, food was withdrawn at 04:00 p.m. One set of animals was decapitated 2 h and another one was decapitated 4 h after treatments, for trunk blood collection for plasma insulin and glucose determination. We also collected the brains 2 h after treatments, for hypothalamic determination of the pIRS-1, pAKT, and pJNK by Western blotting. Another set of rats were submitted to the treatment described above and were perfused 2 h after injections for a qualitative analysis of the pAKT expression in the ARC by immunofluorescence. 

#### 3.2.2. Effect of Central Insulin Administration (ICV) Insulin Administration in Rats Treated with Single or Repeated LPS Injections on Food Intake and Body Weight

One week before the experiment, a cannula was placed in the lateral ventricle of the rats that were then treated as previously described (6 Saline, 5 Saline + 1 LPS, and 6 LPS). On the day of the experiment, at 04:00 p.m., food was withdrawn and the animals were weighed and received the last injection (saline or LPS). Forty-five min after the saline or LPS injection, half of each group received an ICV injection of vehicle (saline, 5 μL) or insulin (12 μM in 5 μL; Sigma, St. Louis, MO, USA) and after 30 min food was reoffered (*n* = 6–8/group). Food consumption and body weight changes were assessed 2 and 14 h after food replacement, respectively. 

#### 3.2.3. Effect of Short and Prolonged LPS Treatment on Insulin Signaling in GT1-7 Cells

To address whether neuronal insulin signaling is affected by short (30′) or prolonged (24 h) LPS treatment, we performed in vitro experiments using the mouse cell line GT1–7 (kindly provided by Pamela Mellon, University of California, San Diego, CA, USA), derived originally from immortalized GnRH hypothalamic neurons. These cells were shown to be responsive to both insulin and LPS treatment [34]. The cells were cultured in Dulbecco’s modified Eagle medium (DMEM; Gibco-Invitrogen, Carlsbad, CA, USA), containing 10% fetal bovine serum and penicillin-streptomycin and maintained at 37 °C in 5% CO_2_. In Vitro experiments were performed in 3–4 different assays. GT1-7 cells were subjected to experimental conditions after 3 days in culture, when the cells were 90% confluent. 4 h before treatment, the cells were kept in serum free medium and then treated with vehicle (sterile PBS), LPS (1 μg/mL) for 30 min, insulin (100 nM/mL) and LPS (1 μg/mL) for 30 min, followed by insulin (100 nM/mL) for 30 min. Another set of GT1-7 cells culture was treated with vehicle or LPS (1 μg/mL) for 24 h, followed by treatment with vehicle or insulin (100 nM/mL) for 30 min. At the end of the incubations, cells were harvested and Western blottings for AKT, pAKT, and GAPDH proteins were performed. To qualitatively evaluate the pattern of expression/distribution of the insulin-induced expression of the transcription factor forkhead box protein O1 (FoxO1) in LPS treated cells, GT1-7 cells were cultured overnight on glass coverslips coated with poly-l-Lysine for immunocytochemistry.

#### 3.2.4. Effect of JNK Inhibition on Food Intake and Body Weight Measurements after ICV Insulin Administration in Rats Treated with Single or Repeated LPS Injections.

Rats implanted with a cannula in the lateral ventricle (*n* = 6–8/group) were assigned into the experimental groups (6 Saline, 1 LPS and 6 LPS) as described above. On the day of the experiment, food was removed at 04:00 p.m. and the rats from each group were ICV pre-treated with vehicle (saline, 5 μL) or JNK inhibitor (20 μM in 5 μL; Tocris, Ellisville, MO, USA), followed 105 min after by vehicle (saline, 5 μL) or insulin (12 μM in 5 μL) ICV injection. Food was reoffered 30 min after vehicle or insulin injection. Food consumption and body weight gain were assessed 2 and 14 h after food replacement, respectively.

#### 3.2.5. Effects of JNK Inhibition on Hypothalamic Insulin Signaling in Rats Treated with Repeated LPS Injection

To evaluate whether JNK inhibition might restore the insulin signaling in the hypothalamus of 6 LPS treated rats, animals (*n* = 6–8/group) were implanted with a cannula in the lateral ventricle. On the 6th day, at 04:00 p.m. the rats received the last IP injection of LPS and 30 min after they received an ICV injection of vehicle (0.2% Dimethyl sulfoxide (DMSO) in 5 μL saline) or JNK inhibitor (20 μM in 5 μL; Tocris Co.: Ellisville, MO, USA). Thereafter, the rats received an ICV injection of vehicle or insulin 105 min after saline or LPS injection. Fifteen min after vehicle or insulin injection, the animals were decapitated and the brains were harvested for hypothalamic pIRS-1 and pAkt measurements by Western blotting.

### 3.3. Cannula Implantation in the Lateral Ventricle 

Rats were anesthetized with a mixture of ketamine (60 mg/kg) and xylazine (7.5 mg/kg) at a volume of 0.1 mL/100 g and placed in a stereotaxic instrument (model 900, David Kopf Instruments: Tujunga, CA, USA). A stainless-steel guide cannula (10 mm) was implanted into the right lateral ventricle of the brain. We used the following stereotaxic coordinates: AP = −0.6 mm, LL = −1.5 mm, and depth = −3.6 mm from bregma. The cannula was held in place with two stainless-steel screws and dental acrylic resin on the skull. To prevent occlusion of the guide cannula, a 30-gauge metal wire filled the cannula. After surgery, the rats received a prophylactic injection of penicillin (50,000 U, intramuscular). The placement of the cannula was confirmed at the end of the experiment by histological analysis.

### 3.4. Western Blotting Analysis of Mediobasal Hypothalamus

The mediobasal hypothalamic protein was extracted using Triton-X 100 (1%), Tris-HCl pH 7.4 (100 mM), sodium pirofosfate (100 mM), sodium fluoride (100 mM), EDTA (10 mM), sodium ortovanadate (10 mM), PMSF (2 mM), aprotinin (0.2 mg/mL), and leupeptin (0.2 mg/mL), at 4 °C, 15,000× *g* for 40 min. Aliquots of the lysates containing 50 μg of protein were denatured in Laemmli sample buffer (6% SDS, 30% glycerol, 0.02% bromophenol blue, 200 mm Tris-HCl (pH 6.8), and 250 mm mercaptoethanol), at 95 °C for 5 min. Samples were blotted onto a nitrocellulose membrane. Nonspecific binding was prevented by immersing the membranes in blocking buffer (10% BSA in Tris-buffered saline-Tween 20, TBS-T) for 90 min at room temperature. The membranes were then exposed overnight to the primary antibodies: rabbit anti-IRS-1 (1:4000, Cell Signaling # 2390); rabbit anti-phospho IRS-1 tyr1222 (1:4000, Cell Signaling # 3066), rabbit anti β-actin (1:1000, Cell Signaling # 8457), rabbit anti-AKT (1:4000, Cell Signaling # 4691), rabbit anti-phospho AKT S473 (1:4000, Cell signaling # 9271), rabbit anti-JNK (1:1000, Cell Signaling # 9252), and rabbit anti-phospho JNK (1:10,000, Cell Signaling # 9251). The blots were rinsed in TBS-T and then incubated with horseradish peroxidase (HRP) conjugated anti-rabbit antibody (1:5000, Cell Signaling # 7074) for one hour at room temperature. Antibody-antigen complexes were visualized by detecting enhanced chemiluminescence using an ECL detection system (Amersham Biosciences) on the digital images using the Quantity One 4.5.0 software (Bio-Rad: Hercules, CA, USA). To normalize pIRS and pAKT expression, total receptor substrate-1 (IRS1) and total AKT were used, respectively. B-actin was used to normalize JNK and pJNK expression. Because we have used antibodies from a rabbit host, total and phosphorylated protein expression were determined in different blots in the same running.

### 3.5. Western Blotting Analysis in Cell Culture

Cells were lysed in ice-cold lysis buffer (25 mM Tris pH 8.0; 1.5 mM EGTA, 0.5 mM EDTA, protease inhibitor cocktail, and Triton X-100 1%). The total protein concentration was determined with the Bradford reagent (Bio-Rad: Hercules, CA, USA). Equivalent amounts of proteins (15 μg/well) were then separated by 10% SDS-PAGE and transferred to nitrocellulose membranes (Bio-Rad). The membranes were subsequently immunoblotted with the appropriate primary antibody at 4 °C overnight (rabbit anti-AKT (1:4000, Cell Signaling # 4691); rabbit anti-phospho AKT S473 (1:4000, Cell signaling # 9271); rabbit anti-phospho AKT T308 (1:4000, Cell signaling # 9275)) and then incubated with HRP conjugated secondary anti-mouse or anti-rabbit antibody (1:4000, Jackson Immuno Research: West Grove, PA, USA). Antibody-antigen complexes were visualized by detecting enhanced chemiluminescence using the ECL detection system (Thermo Scientific: Waltham, MA, USA) on digital images (Bio-Rad). Equal protein loading was assessed by the expression of GAPDH (rabbit anti-GAPDH (1:4000, Cell signaling # 5174)). Total and phosphorylated protein expression were determined in different blots in the same running.

### 3.6. Immunocytochemistry for FoxO1-Dapi

Glass coverslips containing the GT1-7 cells from each experimental group were rinsed with ice-cold PBS and fixed using 4% paraformaldehyde in PBS pH 7.4, at room temperature. After fixation, the cells were rinsed with PBS containing 0.1% Triton-X for permeabilization. After rinsing, the cells were incubated for 30 min in a blocking solution of 1% BSA in PBS containing 0.1% Triton-X. Cells were incubated overnight at 4 °C with the primary antibody (1:5000; rabbit anti-FoxO1, Cell signaling # 2880). After rinsing, the cells were incubated with donkey anti-rabbit IgG conjugated with Alexa 488 secondary antibody (1:500; Jackson Immuno Research: West Grove, PA, USA). Slides were then mounted using a medium containing DAPI (DNA staining, red). Photomicrographs were acquired using a Leica confocal laser scanning microscope. The immunoreactive structures were excited using argon or helium-neon green lasers with the excitation and barrier filters set for the fluorochrome used (green or red). Images showing the fluorescence were obtained from sequentially acquired images of slices excited by the laser.

### 3.7. Plasma Insulin and Glucose Determination

Plasma insulin and glucose concentrations were measured by radioimmunoassay (Millipore: Billerica, MA, USA) and the glucose oxidase method (Doles Regents: Goiania, GO, Brazil), respectively, using commercial kits according to the manufacturer’s protocol.

### 3.8. Immunofluorescence for pAKT

Sections were rinsed with 0.1 M PBS and incubated for 48 h at room temperature with rabbit anti-phospho AKT (1:4000, Cell signaling # 9275). After rinsing, sections were incubated with donkey anti-rabbit IgG conjugated with Alexa 488 secondary antibody (1:400; Jackson Immuno Research: West Grove, PA, USA). Finally, the sections were coverslipped with Fluoromont-G mounting medium (Southern Biotechnology Associates: Birmingham, AL, USA). Photomicrographs were acquired using a Leica confocal laser scanning microscope. The immunoreactive structures were excited using argon or helium-neon green lasers with the excitation and barrier filters set for the fluorochrome used (green). Images showing the fluorescence were obtained from sequentially acquired images of slices excited by the laser.

### 3.9. Statistical Analysis

Results were expressed as means ± SEM and were analyzed using the Software Statistica^®^ (Software Statistica^®^ 10, Tulsa, OK, USA). One-way analysis of variance (ANOVA) followed by the Fisher post hoc test were used to analyze the experiments with single or repeated LPS treatments. Two-way ANOVA, followed by the Fisher post hoc test, were used to analyze the experiment of food intake and body weight gain with single or repeated LPS treatment followed by ICV insulin administration. We used three-way ANOVA, followed by the Fisher post hoc test, to analyze the experiments with single or repeated LPS treatments, JNK inhibitor, and insulin injection. Differences were accepted as significant at *p* < 0.05.

## 4. Conclusions

The main finding of our study is that hypothalamic insulin resistance, induced by an inflammatory challenge after repeated exposure to endotoxins, can be observed in the absence of increased body weight gain. We used a model of 6 days of prolonged low-grade inflammation to cause hypothalamic insulin resistance without body weight changes. The maintenance of this scenario could advance to obesity and diabetes, as shown by Cani and coworkers, which used 4 weeks of endotoxemia to induce glucose homeostasis disturbance, including peripheral insulin resistance, associated with increased body weight [38]. The additive effects of hypothalamic and peripheral insulin resistance during nutrient overload are the key hallmarks of obesity, which could be caused in part by metabolic endotoxemia. The caveat of the present study is that the peripheral effects of insulin were not investigated to assess if central resistance is coupled to systemic resistance to this hormone. Because both peripheral and central insulin resistance are features of obesity, future studies driven to investigate the time course of peripheral insulin effects on metabolism during prolonged endotoxemia must be performed. Our data indicate that alleviation of low-grade inflammation, as a therapeutic target, may help to restore insulin actions in the hypothalamus and the effects on feeding behavior.

It is also important to point out that the reduced metabolism that leads to the increased body weight during the low-grade inflammation is not observed in the more intense inflammatory process such as that seen in individuals or experimental animal models suffering from different cancers. In fact, it is known that severe inflammation induced by cancers induces an opposite metabolic profile known as cachexia [39,40].

Other central inflammatory processes, such as Alzheimer’s disease, also leads to the development of insensitivity to insulin actions and defects in learning and memory [41]. It is crucial to understand the mechanisms underlying impaired insulin signaling during inflammatory processes, to advance in the treatment of obesity and to help alleviate people suffering from degenerative disorders associated with inflammatory markers in the brain. In this context, our study reveals that the inhibition of brain JNK could be used as an intervention approach against obesity and its comorbidities.

## Abbreviations

LPS	lipopolysaccharide
IRS-1	insulin receptor substrate 1
PI3K	phosphoinositide 3- kinase
JNK	Jun NH_2_-terminal kinase
AKT	Protein kinase B
GAPDH	Glyceraldehyde 3-phosphate dehydrogenase
β-actin	Beta-actin
FoxO1	Forkhead box protein O1
SP600125	JNK inhibitor
